# Coxalgia or Hip-Disease

**Published:** 1860-08

**Authors:** 


Coxalgia or Hip-Disease.

In recording the proceedings of the American Medical Association in the July No. of the *Examiner*, we alluded to the paper of Dr. Sayre of New York, on Coxalgia, read and discussed in the Surgical Section of the Association. We then stated briefly the positions assumed in the paper or report, but could not give a summary of the discussion, being engaged in another section at the time it took place. We are now happily enabled to supply the omission by copying the following report from the *American Medical Times*, of New York, for July 14th, and 21st.

"Dr. Lewis A. Sayre of New York, as chairman of the committee appointed at the last meeting of the Association to report on *Morbus Coxarius, and the Surgical pathology of Articular Inflammation generally*, stated that he had prepared a paper referring only to the first branch of the subject, preferring to leave the rest for a future time.

"The present report embraces the pathology, causes, and symptoms of the disease, together with the history of many cases in detail illustrative of the plan, and principle proposed in the treatment of its various stages. Also, a complete collection, in tabulated form, of every case of exsection that had been performed up to the present time—many of which had not been before reported—with a brief history of the same, including the age, sex, cause, condition, time, and mode of treatment; and the result, with the name of the operator, with mention of the record for reference. Also, a full and minute description and engraving of a new instrument, devised by him, for the mechanical treatment of this disease in its earlier stages; an explanation of the principles upon which it was constructed, its mode of application, and result of the treatment illustrated by cases, and photographic drawings taken from life.

"The disease was divided into three stages—first, second, and third. In the first stage, local depletion was advised, together with the removal of all pressure from the synovial surfaces, by means of the instrument referred to. Issues and setons were ignored, and the reasons given therefor. In the second stage, when the effusion was very great and showed no signs of being absorbed by ordinary means, puncture was insisted upon, to be followed by the application of the splint. Cases were cited to show the propriety and harmlessness of this practice if properly performed, not only as a means of relieving the patient, but of arriving at a diagnosis. He maintained that the advantage thus gained by opening the joint was more than counterbalanced by an after risk.

"In the third stage, when the synovial membrane was destroyed, the cartilage of incrustation eroded, and there were positive evidences of bony crepitus present, the operation of *exsection* was strongly urged.

"If such an operation were performed before the acetabulum had become perforated and the system exhausted by hectic fever, there was every prospect of a final recovery; and that within a very few months, with but very slight deformity, and almost perfect motion. Various examples of the benefit of such treatment were given in detail, and the report closed with a tabular review of seventy-two cases of exsection of this joint. Of these operations fifty-eight were performed for caries; forty-four recovered with more or less perfect motion, and the remaining fourteen died; seven from exhaustion, the acetabulum being perforated and the system being broke down by gangrene; two from psoas abscess; three from insufficient removal of the disease; one from fracture; and in one the cause of death not stated. Of the remaining fourteen operations eleven was performed for gun shot wounds, only two recovering; one for fracture; and in the other two the reason for operating was not stated.

"Dr. KRACKOWITZER, of N. Y., asked wherein any splint mentioned differed from the one known as Dr. Davis's, and which of the two had been first in use?

"Dr. SAYRE, in reply, stated that he had seen Dr. Davis's splint before his own was manufactured, and had expressed to that gentleman his disapprobation of the means used for extension, and that he (Dr. S.) had afterwards set to work to construct one that answered, as he thought, the purpose better. He further remarked, that in Dr. Davis's splint there was, in place of the ratchet and cog, a simple hinge arrangement, which was incapable of regulating extension. This he considered a very important point to be looked after, inasmuch as a child would grow fully three or four inches every year, and it was necessary, when the instrument was worn for any considerable length of time, that the means of extension should be so regulated as to meet all the requirements of the case. Dr. S. maintained that in his modification this principle was fully carried out.

"Dr. CROSBY remarked, that the subject of the treatment of hip-joint was a very interesting one to him, more particularly that part of it which referred to the opening of the joint. He thought that the proper time of performing such an operation was a matter well worth discussing.

"Dr. SAYRE, in this connection, stated, that if the joint was fully distended so as to give the peculiar deformity referred to in the second stage of the disease, where the limb was apparently lengthened, flexed, abducted, and everted, and with no signs of the disappearance of the effusion, he would puncture the joint and afterwards apply the splint. The earlier such an operation was performed the better it was for the patient. If, on the other hand, there was good reasons to suppose the existence of sero-purulent matter in the joint, as shown by the long continuence of the disease, general emaciation of the patient, and hectic, a free incision should be resorted to, taking care that no pouch be left.

"Dr. Mussey, of Ohio, asked Dr. Sayre what was the guide for making the puncture.

"Dr. Sayre stated that the puncture was made just behind and above the trochanter major; the depth at which the insturument entered varied with the amount of fat deposited in the sub-tegumentary tissue. In answer to a question from Dr. Atlee, Dr. S. remarked that if the character of the fluid was found after puncture to be sero-purulent, the puncture would be converted into a free incision; if it was then found that the disease had progressed still farther, that the bone had been left bare, all that remained to be done was exsection of the diseased portion. The after treatment consisted in keeping any resulting inflammation in check.

"Dr. Crosby stated, that in a case in which he performed puncture, he first made an incision through the skin and areolar tissue behind the trochanter down to the muscle; then separating the fibres of the same with a director, he ascertained, by the motion of the instrument, the extent of the distension. A trocar was introduced, and synovia and pus escaping, the incision was enlarged in the same manner as referred to by Dr. Sayre. The case treated in this way recovered in a surprisingly short space of time, the patient walking about three months after the incision was made. After the operation, all that remained to be done was to approximate the edges of the wound by adhesive straps, the lower portion being kept open by the introduction of a tent.

"Dr. Hyde asked Dr. Sayre's experience in reference to the opening of other joints.

"Dr. Sayre replied, that he had opened the ankle and elbow joints repeatedly; had followed the same general principle, and had obtained like good results. In reference to the treatment of the early stage of the disease, Dr. Sayre stated that Dr. March, of Albany, had some years before constructed a splint for the purpose of keeping the parts at rest, and preventing any friction or undue pressure of the two inflamed synovial surfaces upon each other. He believed that Dr. M. was the first one who advocated that plan of treatment, and he desired very much to hear that gentleman's experience.

"Dr. Alden March, of Albany, next made in substance the following remarks :—It is true, a few years ago, I brought this subject before this association, and Dr. Sayre has given a faithful account of the views I entertained at that time. The principle of treatment applied more especially to the early stages of the disease, and consisted in keeping the parts in a state of quiescence and in removing all undue pressure. As long ago as the days of Dr. Physic of Philadelphia, a splint was employed in the treatment of this disease. His (Dr. P.'s) idea was simply to prevent motion of the parts, without extension or counter-extension. In 1839, Dr. Wm. Harris published, in the *Philadelphia Medical Examiner*, four cases of morbus coxarius, treated by himself, with extension and counter-extension ; but made no allusion to the pathological condition of the joint structures involved upon which he founded his treatment. My attention was directed to the investigatien of the pathological condition of the most common and destructive form of hip disease as early as the year 1845 or '46. At the session of this Association held in Boston, 1849, at the office of Prof. J. B. S. Jackson, and in his presence, together with some twenty-five or thirty other distinguished surgeons and pathologists, I exhibited several specimens of morbus coxarius, and endeavored to explain the destructive process of this terrible disease. Where two inflamed surfaces rub upon each other, or where undue pressure is made on the tender and inflamed parts, and continued for some time, necrosis and more or less destruction of the joint is pretty sure to follow. The only way to remedy such evil effects was to remove the cause by taking off the pressure. To this end I constructed a rude apparatus, and brought it before the Association at its session in New York, 1853. It consisted simply of a long splint, broader above than below, to which a foot-piece was attached, and a perineal and circular strap or belt. This long outside splint extended from the sole of the foot to a point on the side nearly opposite to the nipple; and at the part opposite to the trochanter major, there was a fenestrum or opening by which all lateral pressure was removed from that projecting point of bone, and consequently from the acetabulum. In regard to the results of this plan of treatment, I find them fully corroborated by the experience of Drs. Sayre and Davis

"But to go further; with regard to the operative part—to the opening of the hip-joint, I must confess I have had very little experience. I have two specimens in my museum of heads of femurs which were necrosed, and were worked out spontaneously. In both instances the patients recovered, and, I believe, are still living, in the enjoyment of good health. I have opened the ankle, knee, and elbow-joints not unfrequently; but I do not remember to have opened the hip-joint more than twice. In one instance, I failed to reach the effusion; but, in process of time, the necrosed bone worked through the opening made; that young man is now alive, and in good health. The mother, at the time of the operation, thought I was too cruel, and in a few days sent for another physician in the neighborhood, who said that I was mistaken in my diagnosis, and that it was nothing but a case of rheumatism.

"He stated, in conclusion, that when his apparatus was first brought forward, he was pretty severely criticised in reference to the supposed ill effects from confinement.

"In Dr. Sayre's apparatus, this confinement, after the acute character of the disease had subsided, was unnecessary, and it was consequently more desirable on that account, as a valuable means of cure. He was glad to see efforts made to improve the treatment of a disease so common, and heretofore so destructive to limb, if not to life; and, if he had been the humble agent in directing the attention of the profession to its mechanical treatment, on true *pathological* and *philosophical* principles, he felt as though he had not in vain devoted many studious hours to this interesting and important subject.

"Dr. Hubbard, of New Hampshire, stated that he had a case of hip-disease which had been managed upon what he considered the conservative principle, where the abcess was allowed to burst. The patient was afterwards placed upon March's splint for three months and a half. As the result of that treatment, the inflammation subsided, and the general health of the patient very much improved, so much so, that it was very desirable to get him up and about. Just at that time Dr. Sayre's report come to hand, and it struck Dr. H. that it was just the instrument that was applicable to that case. Accordingly he sent a measure, and was soon supplied with the apparatus. The splint was first applied in the afternoon with a slight amount of extension, which was increased the following morning. On the following morning the patients clothes were put on him, and he was assisted to walk to the window and sit in a chair, where, at the time of making this report, he still remained. The child is some six or seven years of age, and in testimony of the good effects of the treatment, desired Dr. H. to return his sincere thanks to Dr. Sayre for his instrument. The speaker expressed himself as entirely satisfied with the result of the case, and intended at the very first opportunity again to test the advantage of the instrument.

"Dr. Willard Parker, of N. Y., remarked, in relation to the treatment of the disease in question, that inasmuch as it occurred in scrofulous children, the constitution was the main thing to be looked after; any local appliances being a secondary matter. The constitutional treatment required was sustaining in its character. If any apparatus could be suggested, by means of which the patient might avail himself of exercise, and at the same time keep the tender surfaces apart, a great point would be gained. It seemed to him that Dr. Sayre's apparatus was the result of an old suggestion, and that due credit, as the prime mover in the affair, should be given to Dr. March. He thought that the principle of treatmnet, as laid down by that gentleman, was a correct one,—the prevention of pressure, and the consequent destruction, not only of the synovial membrane, but the cartilage and bony structure.

In reference to the time for opening joints, he did not think it was a question that had been satisfactorily answered. He had some experience in puncturing knee joints, though he never had occasion to perform such an operation upon the hip. In this connection he thought it necessary only to refer to a single case of the former class, which might be considered as a type of the whole. It was a young boy ten years of age, whom he saw in consultation with a surgeon of New York City. The child at that time had been suffering intense pain for some days in consequence of pressure produced by an accumulation of fluid in the cavity of the joint, which had been the seat of acute synovitis. The pain was so intense, that administration of opium and chloroform was found to be entirely useless, as far as any good effects were concerned.— The question naturally enough came up—What was to be done? It was finally decided that an opening should be made. This was accordingly done by a thumb lancet, when so great was the tension of the parts, that the fluid was forced to the extent of fully two feet from the aperture. The fluid, upon examination, was found to be of the nature and consistency of gelatine. The system soon after became tranquil, and sleep followed the administration of an ordinary anodyne. In the course of time a complete recovery was the result.— He could not see the difference between joints which were already the seat of suppuration, where the synovial membrane and cartilage were destroyed and abscesses in another part of the body. The indication for the evacution of the joint were equally strong in both instances.

"Dr. Atlee, of Pennsylvania, thought it was his duty to give his experience in relation to opening of joints, by citing the following case: The patient was a German servant of his, 18 or 19 years of age, with a highly scrofulous constitution.— He was observed limping about the house apparently in great pain; and on being questioned, he told the doctor that for some days he had suffered from severe pain in his knee-joint. Upon examination the part was found very mnch distended, and his suffering was so intense, that it was evident that immediate relief should be given, or else suppuration would be the result. A small trocar was introduced, and about eight ounces of highly albuminous fluid was drawn off. The relief was immediate, and instead of having him laid up for three or four months, in three weeks he was perfectly recovered. He stated, in conclusion, that previous to being compelled to perform the operation, he had always a prejudice against puncturing knee joints, but the result of this case tended to alter his views in relation to that point.

" Dr. McDowell, of St. Louis, stated that he would have given all he had ever made in his profession, and all he expected to make, if he had known of this instrument when his son had morbus coxarius. He should have punctured the joint early, then have applied the instrument, and would have been rewarded by saving his boy. In reference to opening into the knee joint, he stated that he had performed the operation in four instances. In one case, anchylosis was the result; and in three others no serious damage took place. In conclusion he expressed a determination to follow out the principles of treatment as set forth in the discussion.

"Dr. F. H. Hamilton remarked in relation to the treatment of hip disease, that he had early been instructed with reference to the necessity of confinement, but that experience had since taught him the unsoundness of such a principle. He had come to the conclusion that such confinement was in direct antagonism to another and equally important indication, namely, the restoration of the general health. If the child was past six years of age, this was not a very difficult thing to do. His plan was simply to instruct the parents to obtain crutches that were handsomely made of Malacca Wood, and silver mounted, so that the child would not be ashamed of them, nor throw them aside when out among his playmates.— By the adoption of these means simply, the patient would be tempted to take the requisite amount of exercise. To cases under the age referred to, he thought that Sayre's instrument was very well adapted. In reference to operations upon joints he was convinced that there was not so much to be feared in opening them as in making that opening insufficient. He had resorted to the practice not only with impunity, but was satisfied with the result in every case.

" Dr. James R. Wood, of New York, made in substance the following remarks :—The subject of opening joints has interested me for many years, and the opportunities offered for investigating the subject have been ample. The indiscriminate opening of joints is a very serious matter, but there are instances, where the experienced surgeon, by resorting to this practice, will do great good to his patients and credit to his calling. So great was the horror in reference to injury of the joints in days gone by, that even amputation and ligature of the femoral artery in puncture of the knee-joint has been resorted to by our best surgeons, and that within the last fifteen or twenty years. It was because of the resulting constitutional irritation, that this extreme practice was resorted to. I may be permitted here to offer a few thoughts on the different variety of cases in which the joint may be opened. The first is in those cases of traumatic trouble of the joint, where it is opened by puncture as with a penknife, or as is not unfrequently the case, where this has been done, by a drawing-knife, in the hands of a cooper. This latter accident I have met with several times. These are the cases that were so much dreaded by the older surgeons. Here you have acute inflammation speedily terminating in acute abscess of the joint, and the sooner you allow the matter to escape by a free opening the better it will be for the patient; for by so doing, you escape the constitutional irritation and its consequences also, the toxæmic effect from the absorption of matter. Again, as in the case related by Dr. Atlee, where you have the joint filling rapidly with serum, the result of a different grade of inflammation of the synovial membrane, producing excessive distension, excruciating pain, and consequent constitutional symptoms, because of the want of the elasticity of the tissues encroached upon, you are to make a small puncture as you would in the case of accumulation or serum, or pus in the cavity of the thorax; close the wound at once and the relief is immediate. But let me be understood, that I would not resort to these practices in the cases instanced, unless the usual antiphlogistic treatment had been resorted to. I am convinced that it is good surgery, after they have failed, to open the joint as I have stated. Again, we have another form, and one which is very common, in our large cities; it is the result of a constitutional trouble occurring in the badly fed patients, living in pent-up apartments, where the light of heaven and fresh air are seldom admitted; who are sustained by bad food and begotton by strumous or syphilitic parents. In this class of patients we have the disease called *fungus articuli*, by Sir Benj. Brodie, the old-fashioned *white swelling* of our fathers, no matter whether it occur in the hip, shoulder, elbow, knee, or the spine, it is one and the same disease; and although the surgeon may do much, the *medical* treatment should never be forgotten, for without it all surgical appliances will be of but little avail. Give your patient good air, sea air if you can, plenty of light, out-door exercise as much as practicable, iron, wine, or ale, cream, roast and broiled meats, with blood-gravy, and so forth. In these cases, as a general rule, you have the integrity of the joint destroyed before you are consulted; a very different state of things from that existing in the cases already referred to. The synovial membrane, the cartilage of incrustation, and frequently the bone has succumbed to the peculiar grade of inflammation common to this disease. There is but little pain perhaps, but little heat, in fact the swelling about the joint and incapacity of use are the most prominent symptoms presenting themselves; if you exclude the constitutional trouble of the patient which it tis not worth while to refer to here. As in the first form referred to, you have an abcess, but a very different one; in the first you have an acute, a hot abcess, but here yon have a chronic or a cold abcess. It is in all respects like the psoas abcess which occurs in the groin, or the lumbar in the loins. It is in these cases that I have occasionally opened the joints; but I am sorry to say, that my experience is such as to cause me to do it always with reluctance, and let me say here, Gentlemen, that it is my judgement that the good surgeon will always approach a joint with great deference and hesitancy. For even in this class of cases the majority of the patients whom I have operated upon, and those of my neighbors that have fallen under my observation, have either lost their limbs or their lives. Resection, although appearing much more formidable than the simple puncture of the joint, statistics warrant me in saying, is a very much more safe operation, and the results are very much more favorable.

" Dr. Townsend asked Dr. Sayre whether he would open the abscesses that occur upon the thigh in this disease?

"Dr. SAYRE stated, that by the early use of his apparatus, and by following out the plan of treatment set forth, this complication would not take place. If, however, he should meet with a case where such an abcess existed, he did not see any reason why it should not be treated by a free incision as in any other instances.

"On motion of Dr. Atlee, Dr. Sayre's paper was recommended by the section to the Association for publication in its Transactions. The meeting then adjourned *sine die.*"

